# Prevalence of Malaria Parasitemia and Purchase of Artemisinin-Based Combination Therapies (ACTs) among Drug Shop Clients in Two Regions in Tanzania with ACT Subsidies

**DOI:** 10.1371/journal.pone.0094074

**Published:** 2014-04-14

**Authors:** Melissa A. Briggs, Admirabilis Kalolella, Katia Bruxvoort, Ryan Wiegand, Gerard Lopez, Charles Festo, Pierre Lyaruu, Mitya Kenani, Salim Abdulla, Catherine Goodman, S. Patrick Kachur

**Affiliations:** 1 Malaria Branch, Centers for Disease Control and Prevention, Atlanta, Georgia, United States of America; 2 Ifakara Health Institute, Dar es Salaam, Tanzania; 3 London School of Hygiene and Tropical Medicine, London, United Kingdom; Tulane University School of Public Health and Tropical Medicine, United States of America

## Abstract

**Background:**

Throughout Africa, many people seek care for malaria in private-sector drug shops where diagnostic testing is often unavailable. Recently, subsidized artemisinin-based combination therapies (ACTs), a first-line medication for uncomplicated malaria, were made available in these drug shops in Tanzania. This study assessed the prevalence of malaria among and purchase of ACTs by drug shop clients in the setting of a national ACT subsidy program and sub-national drug shop accreditation program.

**Method and Findings:**

A cross-sectional survey of drug shop clients was performed in two regions in Tanzania, one with a government drug shop accreditation program and one without, from March-May, 2012. Drug shops were randomly sampled from non-urban districts. Shop attendants were interviewed about their education, training, and accreditation status. Clients were interviewed about their symptoms and medication purchases, then underwent a limited physical examination and laboratory testing for malaria. Malaria prevalence and predictors of ACT purchase were assessed using univariate analysis and multiple logistic regression. Amongst 777 clients from 73 drug shops, the prevalence of laboratory-confirmed malaria was 12% (95% CI: 6–18%). Less than a third of clients with malaria had purchased ACTs, and less than a quarter of clients who purchased ACTs tested positive for malaria. Clients were more likely to have purchased ACTs if the participant was <5 years old (aOR: 6.6; 95% CI: 3.9–11.0) or the shop attendant had >5 years, experience (aOR: 2.8; 95% CI: 1.2–6.3). Having malaria was only a predictor of ACT purchase in the region with a drug shop accreditation program (aOR: 3.4; 95% CI: 1.5–7.4).

**Conclusion:**

Malaria is common amongst persons presenting to drug shops with a complaint of fever. The low proportion of persons with malaria purchasing ACTs, and the high proportion of ACTs going to persons without malaria demonstrates a need to better target who receives ACTs in these drug shops.

## Introduction

Malaria is one of the leading causes of death in children under five years of age and accounts for up to 40% of outpatient visits in Tanzania [1], [2]. The World Health Organization (WHO) has recommended that persons with malaria receive effective antimalarial treatment within 24 hours of symptom onset, in order to avoid progression to severe disease [3]. This target has been difficult to achieve in Tanzania, where the proportion of children under five with fever who received a first-line antimalarial within one day of symptom onset was just 26% in 2010 [4].

Studies throughout sub-Saharan Africa have shown that the private sector is commonly utilized as an initial source of treatment for malaria or fever [5]. In addition to private health facilities and clinics, this includes pharmacies, drug shops, and general retail stores that sell medicines. While pharmacies are staffed by licensed pharmacists and are permitted to sell prescription-only-medicines, drug shops can be operated by a wide range of providers and have historically been permitted to sell mostly over-the-counter medicines in Tanzania [6]. In 2010, a household survey in three regions in Tanzania, found that of those who sought care for fever in the prior 14 days, 41% sought care at a pharmacy, drug shop, or general store, whereas only 19% went to a government health facility [7].

Patients may choose to seek care in the private retail sector over the public sector for multiple reasons including convenience (hours open), accessibility (proximity to home), reliability of medication stocks, and ability to negotiate the cost of care [6], [8]–[10]. At the same time, studies have documented multiple substandard practices in these shops such as dispensing medications that do not treat the client’s illness or are ineffective because of widespread resistance, dispensing medicines in insufficient quantities, providing inadequate medication instructions, and even dispensing medicines that are counterfeit or of substandard quality [5],[6],[8],[11]–[13].

The opportunities and risks related to retail sector medicine provision have led to the development of a number of initiatives to strengthen the provision of care in drug shops. In Tanzania, key strategies have included the Accredited Drug Dispensing Outlet (ADDO) program and the Affordable Medicines Facility-malaria (AMFm). The Tanzanian government began implementing the ADDO program in 2003. As part of this initiative, drug shop owners apply to have their shops accredited by the government and then undergo a certification process. Drug shop dispensers must attend a 35 day training course, covering key areas such as family planning, malaria treatment and other common illnesses. Shops certified as ADDOs are permitted to sell a limited range of prescription-only-medicines including the artemisinin-based combination therapy (ACT) artemether-lumefantrine (AL), the first-line option for uncomplicated malaria in Tanzania. The program also aims to use increased regulation and business incentives to support best practices [14],[15]. Between 2003 and 2011, ADDO certification and training was completed in over 3,800 drug shops in 15 out of 21 regions in Tanzania [14].

The AMFm aimed to expand access to ACTs in the public and private sectors. ACTs became first-line for uncomplicated malaria in Tanzania in 2006, due to widespread resistance to chloroquine and sulfadoxine-pyrimethamine (SP). Although ACTs are more effective than these older antimalarials, they were initially up to 10–20 times the price, leading to concern that persons seeking malaria care in the private sector would continue purchasing cheaper medicines that were no longer recommended [16]. Hosted by the Global Fund to Fight AIDS, Tuberculosis and Malaria (Global Fund), AMFm was designed to address this problem, by using negotiated procurement and subsidization of ACTs upon purchase from manufacturers to increase “the affordability, availability, market share and use of quality assured ACTs” in public and private markets [17]–[20]. It was piloted at national scale in seven countries, with AMFm-subsidized ACTs first becoming available in mainland Tanzania in late 2010 [20].

One final intervention which may have influenced the use of the drug shops for malaria management was the roll-out of rapid diagnostic tests (RDTs) for malaria diagnosis. RDTs were rolled out in Tanzanian public health facilities from 2010–2012. All patients are now expected to undergo parasitological diagnosis prior to antimalarial treatment, in accordance with WHO guidelines [21]. However, diagnostic testing in the retail sector was officially prohibited, and fewer than 1% of drug shops stocked RDTs in 2011 [20]. As a result, there has been concern that subsidized ACTs sold in drug shops do not necessarily benefit persons who have malaria [22]–[24]. While baseline and endline outlet surveys conducted under an independent evaluation demonstrated that AMFm was successful at decreasing the price, increasing the availability and increasing the market-share of quality assured ACTs in the Tanzanian private sector, the degree to which subsidized ACTs in the private sector were successfully targeted to those with malaria parasitemia was not assessed [17],[20].

In 2004, a study in one district in Tanzania found a malaria prevalence of 24% amongst drug shop clients presenting with a complaint of malaria or fever [25]. This was higher than the 11% malaria prevalence detected by a contemporaneous random survey of healthy individuals in the same communities, but was not significantly different than the 30% malaria prevalence noted in febrile patients in health facilities in the same district. Only 17% of drug shop clients in that study obtained a medication with antimalarial activity and only 9% purchased SP, the first-line medication for malaria at that time [25]. However, similar data have not been collected since the implementation of ACT subsidies.

Given the questions regarding the performance of drug shops in the management of malaria in Tanzania, this study was designed to assess the current prevalence of malaria in drug shop clients; ACT purchasing patterns among drug shop clients; and predictors of ACT purchase in the setting of ACT subsidies, in areas with and without ADDO certification.

## Methods

### Ethics Statement

All drug shop attendants were provided with a full explanation of the study in the language of their choice, English or Swahili. They were also informed that this study was being performed for educational and policy purposes only, that participation was not required, and that there would be no adverse consequences should they choose not to participate. Written informed consent was required before the drug shop could be enrolled in the study. If consent was not obtained, the research team was not permitted to record information about the drug shop or recruit clients from that shop.

When recruiting clients, research staff again had to explain the purpose of the study, risk and benefits of participation, and freedom to decline participation at any time to all potential participants. Written informed consent was required for all participants over the age of 18. Children under the age of 18 required the written consent of a guardian to participate and assent was requested of children age 7 to 18. Each participant signed two copies of the form, one copy which they were allowed to keep, and one which was kept by the research team as documentation of consent.

All study procedures and consent forms were submitted to and approved by the institutional review boards at the Tanzanian National Medical Research Institute (Dar es Salaam, Tanzania), Ifakara Health Institute (Dar es Salaam, Tanzania), Centers for Disease Control and Prevention (Atlanta, GA, USA), and London School of Hygiene and Tropical Medicine (London, UK).

### Study Design

**Figure 1:**
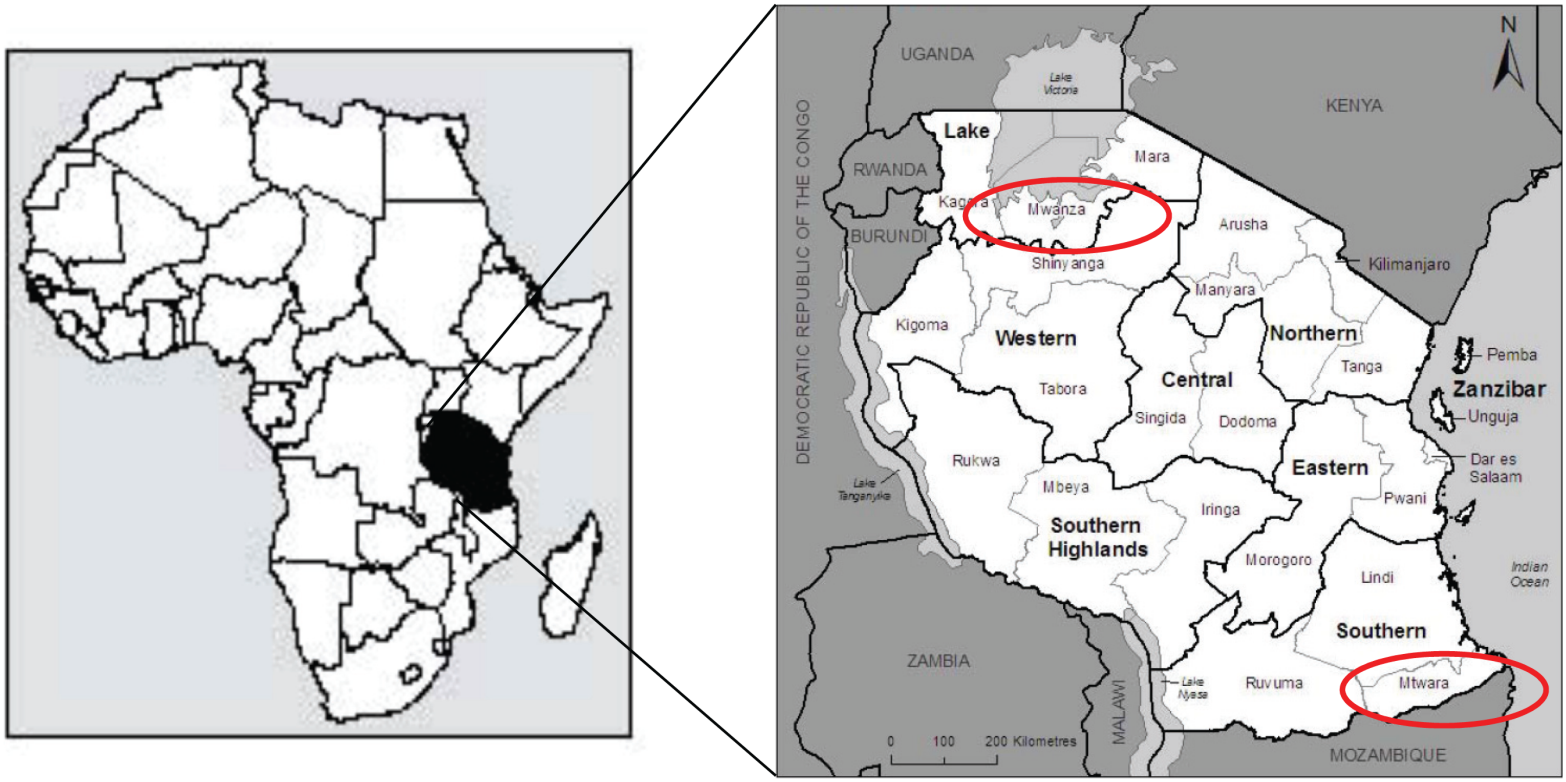
Map showing Mwanza and Mtwara Regions (Tanzania HMIS, 2008). Reference: Tanzania Comission for AIDS (2008). Tanzania HIV/AIDS and Malaria Indicatory Survey 2007–08. Dar es Salaam, Tanzania.

We performed a cross-sectional survey of drug shops in two regions in Tanzania, Mtwara and Mwanza ***(*** ***Figure 1*** ***)***. These regions were selected to correspond with other studies in households, health facilities, drug stores and pharmacies around the same time. Data collection occurred in Mtwara from March 2–26, 2012, and in Mwanza from April 5-May 2, 2012. Both regions experience moderate to high malaria endemicity. Amongst children aged 6–59 months tested for malaria as part of a national household survey during 2011–2012, 17.4% tested positive by RDT and 2% tested positive by microscopy in Mtwara, while in Mwanza, 18.6% tested positive by RDT and 5.4% by microscopy [26]. This can be compared to a nationwide malaria prevalence of 9% by RDT and 4% by microscopy amongst children age 6–59 months in the same survey. At the time of the study, the ADDO program was implemented in the region of Mtwara in 2007 but had not yet been introduced in Mwanza.

### Sampling and Recruitment

Drug shop clients were sampled by stratified, randomized cluster sampling. The sample size was calculated using the expected malaria prevalence in drug shop clients as the primary outcome. Based on a conservative estimated malaria prevalence of 42%, precision of 0.10, power of 80%, and design effect of two, we calculated a required sample size of 187 per stratum in order to measure malaria prevalence with 95% confidence. In order to be able to stratify the data into clients <5 and clients >5 years, we aimed to sample at least 374 clients per region.

The Tanzanian Food and Drug Authority (TFDA) provided lists of all registered drug shops in each region. Drug shops in the three urban districts (Mtwara Municipality in Mtwara; Nyamagana and Ilemela in Mwanza) out of the total 14 districts in the two regions were excluded to better evaluate health practices in the private sector in settings with more limited access to public health facilities. The remaining lists were confirmed with medical and/or pharmaceutical authorities in each district. A fixed number of drug shops was randomly sampled from each district to provide at least 30 shops per region; approximately10 shops per district in Mtwara and six shops per district in Mwanza. At each district, the research team visited all selected drug shops and consented drug shop owners or attendants for participation. Shops that were closed were visited again a second day. If a drug shop was identified as permanently or indefinitely closed, a replacement shop was randomly selected from the list of shops in that district. In one district the total number of shops was too small to allow for replacements, and all available shops were included.

Researchers spent one full day at each shop and approached all clients exiting the shop to assess eligibility for participation after they had completed their purchase. Clients were considered eligible for inclusion if they were suspected to have malaria, defined as having a primary complaint of malaria or fever, or having purchased an antimalarial or anti-pyretic medication. The person who was ill or that person’s guardian had to provide informed consent for participation. If the ill person and/or guardian were not present at the shop and they resided less than 10 km away, they were consented and interviewed at their home. For logistical reasons, clients were excluded if the person receiving the medication was not present at the shop and resided more than 10 km away. Clients were also excluded if the person receiving the medication was <3 months of age, pregnant, or showed signs of severe disease. ACTs are not first-line for any of these groups; as a result persons in these categories were referred directly to the nearest health facility, with transportation provided if necessary.

### Data Collection and Laboratory Studies

Interviews were performed by clinician researchers, all of whom had experience caring for malaria patients in the clinical setting in Tanzania, and who underwent one week of training prior to the study. Data were captured on personal digital assistants (PDAs) that included all questions in both English and Swahili. Drug shop attendant interviews were performed to assess drug shop accreditation and ownership, as well as drug shop attendant education, experience and training. Training referred to any in-service training offered by the government or non-governmental organizations. Client exit interviews included a brief questionnaire to assess presenting symptoms, prior health care seeking behaviors and medications purchased.

Clients also underwent a brief physical examination including axillary blood temperature, measurement of pulse and respirations, and auscultation of the heart and lungs. They then had a finger-stick blood sample taken for laboratory studies, including RDTs (pan-specific ICT Diagnostics, Capetown, South Africa), blood films and dried blood spots. On-site hemoglobin (Hb) tests (HemoCue Hb system) were also performed on children under five years. RDTs and Hb results were read immediately by trained field staff and appropriate treatment was provided, if the patient had not already purchased it. These included age appropriate treatment for uncomplicated malaria and anemia according to the Tanzanian National Guidelines for Diagnosis and Treatment of Malaria, and basic treatments for acute respiratory illness and diarrhea according to guidelines for the Integrated Management of Childhood Illnesses [27],[28]. Blood films were stained and filter paper dried blood spots were prepared by a certified laboratory technologist in the field. The blood films were then transported to a central reference laboratory where they were read by two different laboratory specialists, each blinded to the initial RDT result as well as the other microscopist’s reading. Discordant results were resolved by a third microscopist. In addition, if the final microscopy result differed from the RDT, the filter paper was used to perform a polymerase chain reaction (PCR) test for malaria. PCRs were performed by the CDC laboratory using the Quiagen extraction method, and based on 18 S subunit of *Plasmodium* sp. ribosomal RNA. Persons were defined as positive for malaria if they either had a concordant positive RDT and blood film, or they had a positive PCR for malaria.

### Analysis

Data were analyzed in SAS 9.3 (SAS Institute Inc, Cary, NC). Univariate analysis of drug shop descriptors and client demographics was performed both combined and stratified by region. A wealth index was calculated for participants using a standardized set of 30 questions assessing the quality of the participants’ housing, access to resources, and items owned. The principal component function was then used to categorize participants by wealth quintile. The quintile assigned reflects only relative wealth internal to the study and not overall wealth compared to other Tanzanians.

Frequencies were weighted to account for the proportion of drug shops selected in each district, also accounting for the proportion of shops within each region that were closed and needed to be replaced. Because all persons visiting the shop on the day of the visit were approached for potential inclusion in the study, the number of persons attending each shop was considered self-weighting, and no additional adjustments were made for this in the analysis. Differences between the regions were measured using chi-squared analysis, or Fisher’s exact tests for tables with cell values less than five. All p-values were two-tailed, and a p-value of <0.05 was considered statistically significant.

Predictors of ACT purchase were assessed using multiple logistic regression, including all predictors with a p-value of <0.2 on bivariate analysis or a suspected effect on the outcome based on prior studies or logical effect paths. Initial variables assessed included both drug shop level variables (*region, accreditation status, history of ADDO training, availability of subsidized ACTs, ACT-specific training, any other in-service trainings, drug shop attendant education, drug shop attendant years of experience*) and client-level variables (*age, gender, wealth quintile, head of household education, history of prior care, visit to a health facility before arrival, diagnosis of malaria at the health facility, receipt of prescription at the health facility, chief complaint of fever, fever on exam at the drug shop, pallor, purchase of paracetamol, purchase of antibiotics*). Correlated variables such as region and drug shop accreditation, or visit to health facility and malaria diagnosis at the health facility, were modeled separately. Interactions were also assessed and, if detected, the model was run with and without a variable accounting for the interaction. The final model including primary variables and interactions was chosen based on best model fit taking into account both the Akaike Information Criterion and Schwarz’s Bayesian Information Criterion [29],[30]. Survey procedures were used to account for district level stratification and drug-shop level clustering in all analyses, via Taylor series approximation [31].

## Results

**Figure 2:**
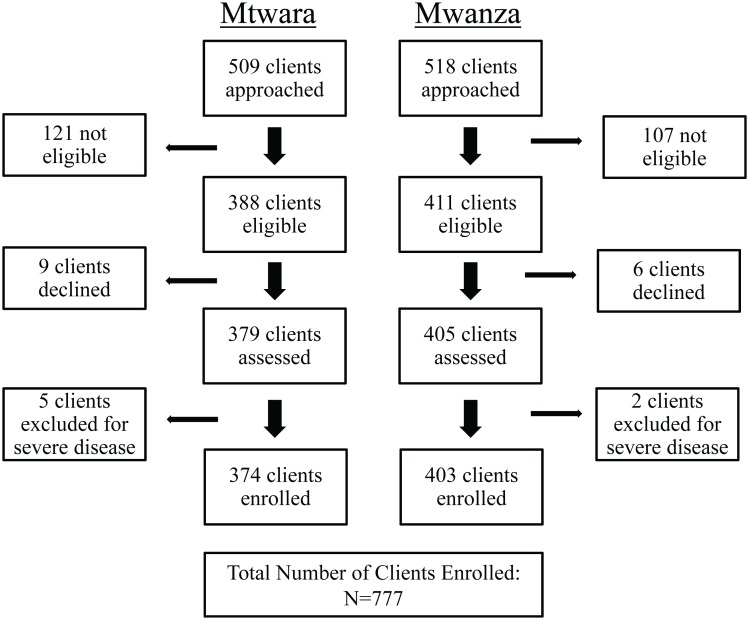
Client Recruitment.

In total 73 drug shops were enrolled, 37 in Mtwara and 36 in Mwanza. The research team approached a total of 1027 clients, 825 of whom were there for malaria or fever. Twenty-six clients were ineligible for participation because they were aged under 3 months (n = 1), pregnant (n = 9), lived more than 10 km from the drug-shop (n = 13), were shopping for a client who was not present, but declined a home visit (n = 2), or did not complete their eligibility assessment (n = 1). Of the remaining 799 eligible participants,15 chose not to consent, 7 were excluded due to signs of severe disease, and 777 were enrolled, 374 in Mtwara and 403 in Mwanza ***(*** ***Figure 2*** ***)***. For forty-two (5%) of these the research team visited the client at their home in order to complete the consent and data collection.

### Drug Shop Characteristics

Drug shop attendants reported that the shop was ADDO certified in 96% of shops in Mtwara and none in Mwanza ***(*** ***Table 1*** ***)*** *.* Availability of subsidized ACTs did not differ significantly between regions, with 100% of shops in Mtwara reporting that they carried subsidized ACTs and 81% in Mwanza. In-service training on ACT use was reported in nearly 59% of shops in Mtwara, and 19% in Mwanza. Less than a third of interviewees reported attending other in-service trainings.

**Table 1:** Drug Shop and Drug Shop Attendant Characteristics Stratified by Region.

Variable, n (%)	Mtwara (n = 37)	Mwanza (n = 36)	*P-*value
**Training and Certification**			
ADDO certification completed*	35 (96%)	0 (0%)	–
Received ADDO training* †	31 (88%)	0 (0%)	–
Sells ACTs that are subsidized by AMFm	37 (100%)	27 (81%)	–
Received ACT-specific training* †	22 (63%)	7 (28%)	0.0181
Received other in-service trainings (not ADDO or ACT)†	12 (31%)	5 (10%)	0.0949
**Attendant education (highest level completed)**	–	–	0.3378
Primary school	5 (14%)	7 (20%)	
Secondary school	14 (39%)	7 (20%)	
Certificate	13 (34%)	15 (42%)	
Diploma or degree	5 (13%)	6 (19%)	
**Attendant experience**	–	–	0.9276
Less than 2 years	5 (15%)	4 (14%)	
2–5 years	12 (35%)	14 (41%)	
Greater than 5 years	17 (49%)	17 (45%)	

† *At least one person working in the shop received the training described.*

* *Weighted proportions differed significantly between regions.*

There was no significant difference between Mtwara and Mwanza in either the highest level of education achieved by the drug shop attendant or the overall years of experience selling medications. Over half of drug shop attendants had completed secondary school or obtained a post-secondary school certificate. Less than 20% had a diploma or degree. Nearly fifty percent of attendants reported having more than five years of experience selling medications in both regions.

### Client Characteristics

Approximately a quarter of all participants were under five years while over half were greater than 15 years old in both Mtwara and Mwanza ***(*** ***Table 2*** ***)***. In both regions, the proportions of males and females were approximately equivalent. Most participants came from households where the head of household had completed primary school, but less than 20% had completed secondary school or post-secondary school education. Over 90% of participants in both regions reported having at least one insecticide-treated bed net in the home.

**Table 2:** Client Demographics and Health-Seeking Behaviors Stratified by Region.

Variable, n (%)	Mtwara (n = 374)	Mwanza (n = 403)	*P-*value
**Demographics**			
Age*	–	–	0.0408
<5 years	74 (20%)	111 (30%)	
5–14 years	47 (13%)	59 (14%)	
≥15 years	253 (68%)	233 (56%)	
Female	177 (48%)	207 (50%)	0.5217
**Head of household education**	–	–	0.2243
Did not complete primary school	70 (19%)	93 (25%)	
Completed primary school	248 (66%)	251 (61%)	
Completed secondary school	52 (15%)	58 (14%)	
Bed-net ownership	347 (93%)	378 (95%)	0.3948
**Wealth Index (calculated)** *			0.5222
Poorest quintile	78 (19%)	75 (19%)	
2^nd^ quintile	62 (15%)	91 (22%)	
3^rd^ quintile	90 (25%)	67 (19%)	
4^th^ quintile	70 (19%)	87 (20%)	
Wealthiest quintile	74 (21%)	83 (21%)	
**Health-Seeking Behaviors**			
Seen at health facility (HF) same day as drug shop visit*	55 (14%)	19 (3%)	0.0001
Diagnosed with malaria at HF*	46 (12%)	14 (3%)	0.0003
Provided prescription at HF*	46 (12%)	16 (3%)	0.0004
Any prior care this illness*	86 (24%)	36 (9%)	<.0001
Prior antimalarial this illness*	34 (9%)	15 (4%)	0.0123

* *Weighted proportions differed significantly between regions.*

Healthcare seeking behaviors differed significantly between the regions, with 24% of clients in Mtwara reporting that they had previously sought care for their current illness, compared to just 9% of clients in Mwanza. In Mtwara, 14% of clients had been to a health facility the same day as their visit, whereas in Mwanza only 3% of clients had. Over 70% of these persons had been diagnosed with malaria and/or provided with a prescription, prior to their visit to the drug shop.

When asked about their symptoms, over 95% of participants reported fever. Approximately half complained of cough or trouble breathing, and a similar amount noted vomiting, diarrhea or abdominal pain. Headache and runny nose were each reported by <10% of clients in each region ***(*** ***Table 3*** ***)***. Fever (temperature >38°Celsius) was present on examination for 17% of participants in Mtwara and 11% of participants in Mwanza. Hb levels were only assessed in participants less than five years of age. In this age group, mild anemia (Hb 7–11 g/dl) was present in 64% of clients in Mtwara and 53% of clients in Mwanza. Only 1% in each region had severe anemia (Hb 5–7 g/dl) and none had life-threatening anemia (Hb <5 g/dl).

**Table 3:** Client Illness History and Exam Findings Stratified by Region.

Variable, n (%)	Mtwara (n = 374)	Mwanza (n = 403)	*P-*value
**Presenting Symptoms**			
Fever	352 (94%)	388 (97%)	0.0669
Cough/trouble breathing*	153 (40%)	211 (55%)	0.0008
Abdominal symptoms	148 (39%)	197 (49%)	0.0711
Headache*	33 (9%)	13 (2%)	0.0003
Runny nose	19 (5%)	22 (5%)	0.9510
**Beginning of symptoms**	–	–	0.5861
Same day	26 (7%)	18 (5%)	
One day before	148 (39%)	134 (36%)	
2–7 days before	178 (48%)	220 (53%)	
>7 days before	22 (6%)	31 (6%)	
**Exam Findings**			
Fever	66 (17%)	33 (11%)	0.0666
Tachycardia*	31 (8%)	6 (1%)	0.0003
Pallor*	9 (3%)	5 (1%)	0.0183
Course breath sounds	37 (9%)	42 (13%)	0.4687
Abdominal tenderness*	42 (11%)	20 (4%)	0.0005
**Hemoglobin Results**	–	–	0.2787
No anemia (Hgb >11g/dl)	27 (34%)	54 (47%)	
Mild anemia (Hgb 7–11g/dl)	46 (64%)	56 (53%)	
Severe anemia (Hgb <7g/dl)	1 (1%)	1 (1%)	

*Proportions differed significantly between regions.

### Prevalence of Malaria Parasitemia

**Figure 3:**
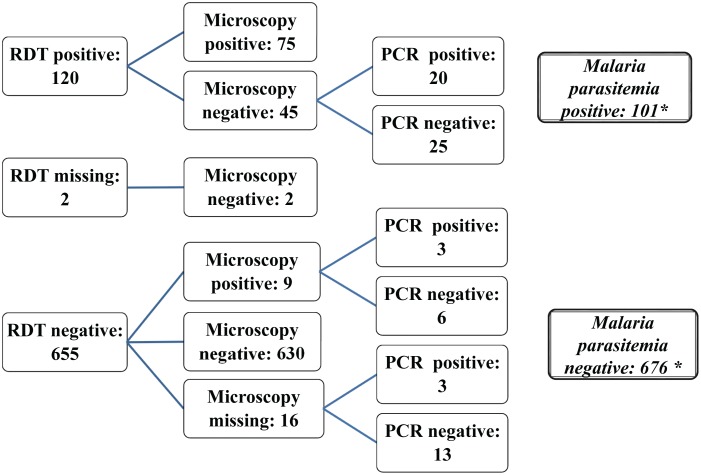
Total Number of Participants with and without Malaria Parasitemia based on Laboratory (RDT, Microscopy and PCR) Results. *Malaria parasitemia positive was defined as either concordant RDT and microscopy positive (75) or PCR positive (26) for discordant results. Malaria parasitemia negative was defined as either microscopy negative without discordance (632) or PCR negative (44) for discordant results.

**Figure 4:**
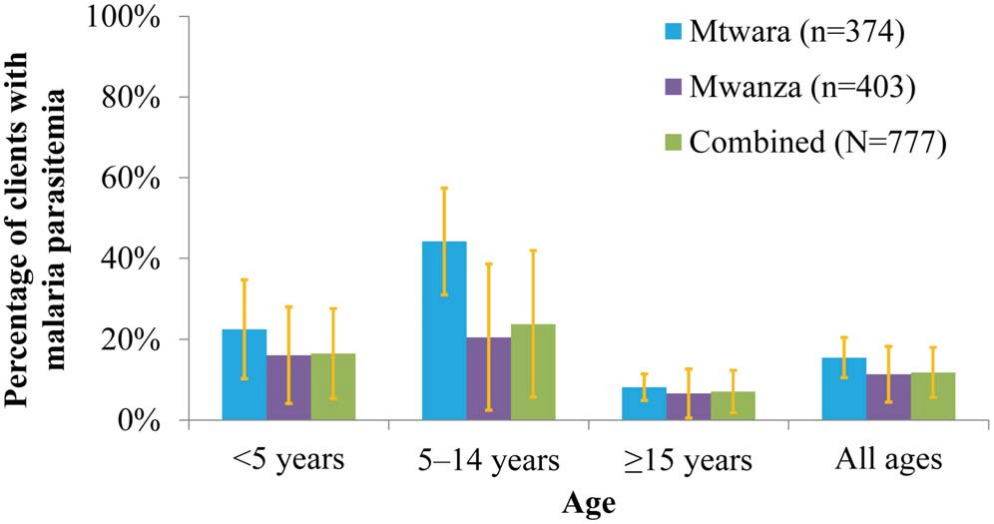
Malaria Parasitemia Prevalence Stratified by Age and Region.

As noted above, malaria parasitemia was determined by a combination of tests including RDT, microscopy and PCR ***(*** ***Figure 3*** ***)***. There were 120 participants with a positive RDT and 84 participants who tested positive for malaria by microscopy. In total 101 out of 777 participants tested positive for malaria parasitemia using the algorithm described above. The prevalence of malaria parasitemia was highest in children aged 5–14 years, with a weighted prevalence of 44% (95% CI: 31–57%) in Mtwara and 21% (95% CI: 2–39%) in Mwanza ***(*** ***Figure 4*** ***)***. There was no significant difference by region in the prevalence of malaria in the other age-groups. Approximately 17% (95% CI: 5–28%) of children under five years and 7% (95% CI: 2–12%) of persons greater than 15 years tested positive for malaria. The overall prevalence of malaria was 12% (95% CI: 6–18%).

### Medication Purchases

Approximately a third of participants had purchased an antimalarial for their symptoms, prior to their laboratory evaluation. In Mtwara, the region with the ADDO-certified shops, nearly a quarter of participants had purchased ACTs, 80% of which were subsidized. Purchase of subsidized ACTs was significantly lower in Mwanza, where just 16% of participants purchased ACTs (p = 0.0106), 60% of which were subsidized. SP was purchased by over 10% of clients in each region, and amodiaquine monotherapy and quinine, were each purchased by less than 5% of clients in each region. None of the participants purchased chloroquine or artemisinin monotherapies. Non-antimalarial medications included paracetamol, which was purchased by 71% of clients in Mtwara and 87% of clients in Mwanza, and antibiotics, which were purchased by 11% of clients in each region. ***Table 4*** presents the medications purchased in each region, stratified by malaria status.

**Table 4:** Proportion of Clients With and Without Malaria Purchasing Antimalarials and Other Medications, Stratified by Region.

Variable, n (%)	MTWARA			MWANZA		
	Clients withmalaria (n = 61)	Clients withoutmalaria (n = 313)	*P*-value	Clients withmalaria (n = 40)	Clients withoutmalaria (n = 363)	*P*-value
**Any Antimalarial** *	37 (60%)	108 (34%)	0.0008	9 (18%)	118 (32%)	0.1230
**ACTs**						
Artemether-lumefantrine (AL)*	26 (42%)	60 (18%)	0.0004	3 (6%)	45 (12%)	0.1614
Subsidized AL*	22 (35%)	52 (15%)	0.0010	2 (5%)	37 (10%)	0.2208
Artesunate amodiaquine (AA)	1 (1%)	5 (2%)	0.7553	1 (2%)	18 (6%)	0.1958
Subsidized AA	0 (0%)	0 (0%)	–	0 (0%)	1 (1%)	–
Any ACT*	27 (44%)	65 (19%)	0.0008	4 (8%)	63 (17%)	0.0396
Any subsidized ACT*	22 (36%)	52 (15%)	00010	2 (5%)	38 (10%)	0.1883
**Non-ACT antimalarials**						
Sulfadoxine-pyrimethamine	8 (14%)	33 (11%)	0.5127	4 (8%)	42 (11%)	0.6415
Amodiaquine	2 (2%)	1 (<1%)	0.0889	0 (0%)	11 (3%)	–
Quinine	0 (0%)	9 (3%)	–	1 (2%)	2 (<1%)	0.1975
**Other medications**						
Paracetamol (acetaminophen)	45 (74%)	220 (70%)	0.6746	33 (84%)	311 (87%)	0.3352
Antibiotic	7 (12%)	35 (11%)	0.7394	6 (10%)	48 (11%)	0.7043

*Proportion differed significantly by malaria status, in at least one region.

### Predictors of ACT Purchase

**Figure 5:**
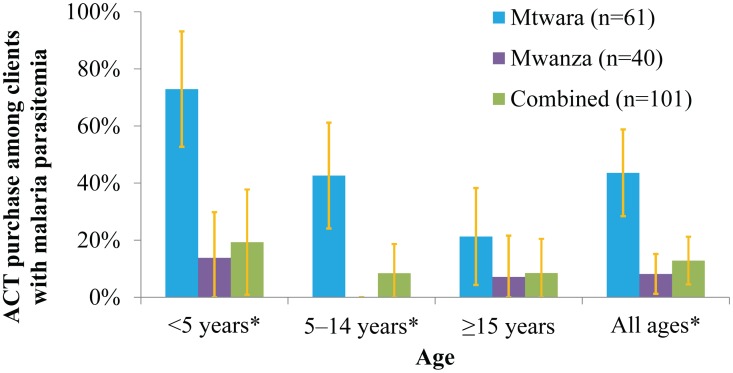
ACT Purchase Among Clients with Malaria Parasitemia Stratified by Age and Region. *Proportions differed significantly between regions.

***Figure 5*** shows the proportion of clients with malaria that purchased ACTs, stratified by age and region. ACT purchase amongst clients with malaria was significantly higher in Mtwara where 44% bought an ACT compared to just 8% in Mwanza (p<0.0001). A higher proportion of children under five with malaria received an ACT than any of the other age groups. This was most pronounced in Mtwara where 73% of children under five with malaria received an ACT.

**Figure 6:**
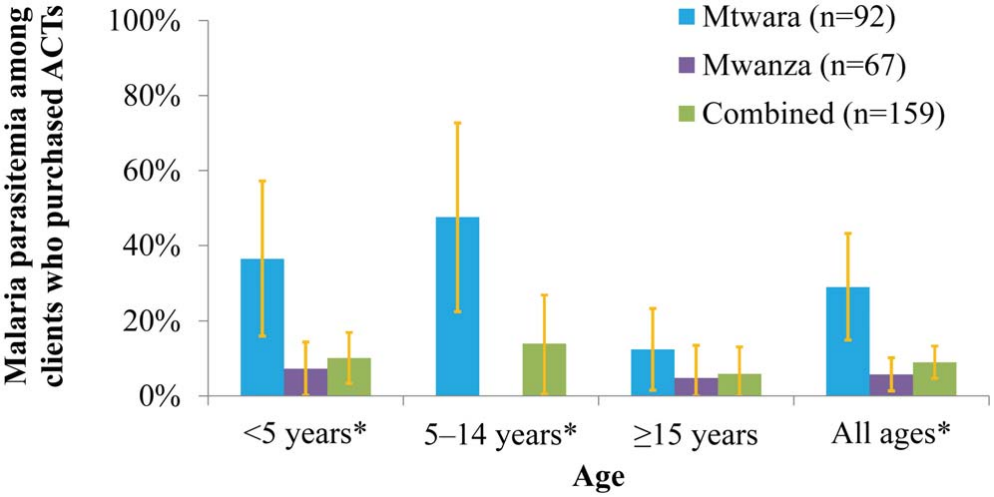
Malaria Parasitemia among Clients Who Purchased ACTs Stratified by Age and Region. *Proportions differed significantly between regions.

**Figure 7:**
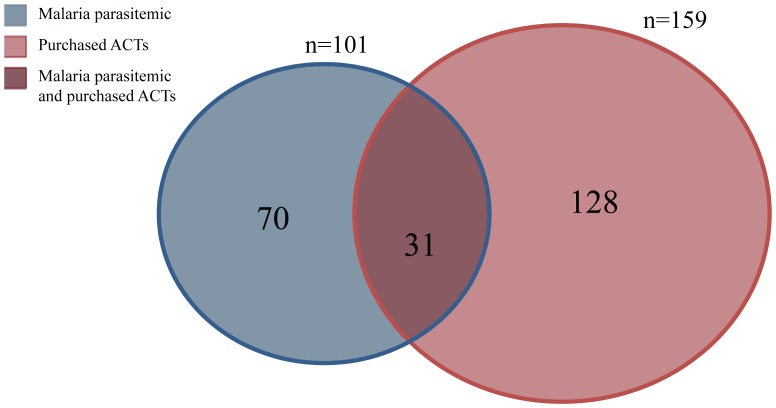
Diagram of Persons with Malaria Parasitemia vs. Persons who Purchased ACTs. *101 (13.0%) persons had malaria parasitemia. 159 (20.5%) persons purchased ACTs. 31 (30.7%) of those with malaria parasitemia purchased ACTs. 128 (80.5%) of those who purchased ACTs did not have malaria parasitemia.

***Figure 6*** shows the prevalence of malaria amongst clients who purchased ACTs. This also differed significantly between regions. In Mtwara, the region with the ADDOs, 29% of the participants that bought ACTs had malaria compared to just 6% in Mwanza (p = 0.0001). In Mtwara, about 37% of children under 5 and 48% of children aged 5–14 years whose parents/guardians bought ACTs had malaria. This was much higher than in Mwanza where only 8% of children under 5 whose parents bought ACTs had malaria. ***Figure 7*** presents a graphic representation of the overlap between patients with malaria and clients purchasing ACTs for their complaint in the two regions combined.

***Table 5*** shows the best fit model for predictors of ACT purchase. Age under five was the most significant predictor with an adjusted odds ratio (aOR) of 6.6 (95% CI: 3.9–11.0). Other significant predictors included being seen at a health facility the day of the visit (aOR: 1.9; 95% CI: 1.0–3.3) and having an attendant with >5 years of experience (aOR: 2.8; 95% CI: 1.2–6.3). Purchasing paracetamol or antibiotics negatively predicted ACT purchase (aOR: 0.3; 95% CI: 0.2–0.4 and aOR 0.4; 95% CI: 0.2–0.9 respectively). Malaria parasitemia was a significant predictor of ACT purchase with an aOR of 1.8 (95% CI: 1.0–3.3). However, there was a significant interaction between malaria parasitemia and region. Stratifying parasitemia by region demonstrated that this was driven entirely by Mtwara, where having malaria parasitemia was a predictor of receiving an ACT with an aOR of 3.4 (95% CI: 1.5–7.4). In contrast, malaria parasitemia was a borderline negative predictor in Mwanza with an aOR of 0.5 (95% CI: 0.2–1.3). Region by itself was not a significant predictor of ACT purchase (aOR: 1.5; 95% CI: 0.8–2.8).

**Table 5:** Predictors of ACT Purchase (n = 763).

Variable	aOR	95% CI	*P*-value
Age (Ref: ≥15 years)			
<5 years	6.6	3.9–11.0	<0.0001
5–14 years	2.7	1.4–5.3	<0.0001
Visit to a health facility first	1.9	1.0–3.3	0.0420
Drug shop attendant experience (Ref: <2 years)			
2–5 years	1.7	0.7–4.1	0.2772
>5 years	2.8	1.2–6.3	0.0146
Bought paracetamol	0.3	0.2–0.4	0.0001
Bought antibiotics	0.4	0.2–0.9	0.0212
Malaria parasitemia/Mtwara	3.4	1.5–7.4	0.0025
Malaria parasitemia/Mwanza	0.5	0.2–1.3	0.1530

## Discussion

This study assessed ACT purchase in the private sector in the setting of a national ACT subsidy program and sub-national drug-shop certification and training program. As in previous studies, over three-quarters of clients in both regions presented to the drug shop as their initial source of care. Overall 12% of drug shop clients with a chief complaint of malaria or fever had malaria parasitemia. Even in the setting of ACT subsidies, less than a third of all drug shop clients with malaria obtained ACTs, and most of the ACTs that were purchased were bought for clients that did not have malaria.

Surveys of malaria parasitemia prevalence were performed in health facilities around the same time that this study was being performed [32]. Malaria prevalence by microscopy among persons seeking care for malaria was nearly equivalent (11%) in drug shops and health facilities in Mwanza, where both surveys were conducted in April–May 2012. In Mtwara, parasitemia prevalence was twice as high at facilities (15% at drug shops, v. 32% at health facilities). However, the health facility survey in Mtwara was performed towards the end of the rainy season (June–July 2012), when higher malaria parasite prevalence would be expected.

In this study, drug shop clients under five years old were more likely to have received ACTs than older clients, even when controlling for prior health care seeking. This likely indicates a perception of increased risk in this age group, which might be appropriate given the higher risk of progression to severe disease in children less than 5 years. However, in both regions malaria test positivity was highest in school-age children (age 5–14). These children were three times less likely than their younger counterparts to receive the correct medicine if they tested positive for malaria. This pattern of malaria prevalence, wherein the prevalence was highest in children age 5–14 was also seen in the contemporaneous health facility survey mentioned above [32].

This study also demonstrated a difference in ACT targeting between the two regions, wherein clients with malaria were more likely to receive ACTs in Mtwara, the region where the ADDO program had been implemented, than in Mwanza, the region with uncertified drug shops. Unfortunately, the cross-sectional design of this study did not allow for full evaluation of the effect of the ADDO program on provider practices. In addition, ADDO certification, training, and region were all highly correlated so they could not be tested in a composite model to look at their independent effects. We did find that in addition to receiving ADDO certification, drug shop attendants were more likely to have received training on subsidized ACTs in Mtwara as compared to Mwanza. Although this was not found to be a significant predictor in the model, it is clear that some component of either drug shop attendant practice or client behavior led to higher ACT sales in Mtwara, compared to Mwanza even after controlling for availability of subsidized drugs, prior care, income, and other factors measured in this study.

One limitation of this study is that it was completed in registered drug shops, based on lists provided by the TFDA. The findings in this study cannot be generalized to unregistered shops and other retail stores where practices may differ. In addition, it is possible drug shop attendants or clients that participated in this study may have altered their behavior due to the presence of the study team. Although the researchers remained outside of the drug shop and only approached clients after their purchase had been completed, it is possible that community members knew that medical providers were present providing free diagnostic tests and treatment for malaria and this may have altered typical patterns of drug shop attendance or drug purchasing behaviors. We tried to limit these biases by asking drug shop attendants and participants not to share any specific details about the study with others, and by limiting the time spent at each shop to one day.

Even with these limitations, this is currently the only study reporting antimalarial use in the private sector in the setting of subsidized ACTs and in the absence of malaria diagnostic testing. Two recent studies in Uganda and Cameroon provide additional evidence on parasite prevalence and antimalarial purchase from medicine retailers, in settings without ACT subsidies [33],[34]. The proportion of clients that tested positive for malaria parasitemia was higher in both studies compared to the Tanzania results: 27% by blood slide in Uganda, and 24% by RDT in Cameroon. Amongst drug shop clients who tested positive for malaria the percentage receiving an ACT in those studies was similar in Uganda (32%), but was actually higher in Cameroon (48%), though this may have been in part due to the inclusion of pharmacies as well as drug stores in the Cameroon study [34].

The high level of paracetamol use for malaria/fever treatment in this study was likely influenced by its low price, which is typically 0.03–0.06 USD per tablet. However, the persistent popularity of non-ACT antimalarials is harder to explain by price alone. The AMFm program was based on the idea that if ACTs were available at the price of other inexpensive antimalarials, they would crowd out other medications and would lead to increased access to these essential medicines in rural communities where access to public health facilities can be difficult [16],[24]. In fact, an independent evaluation of the AMFm program overall found it to be quite a success by certain measures. In Tanzania and multiple other countries, baseline and endline surveys revealed that the subsidy was successful at decreasing the price, increasing the availability and increasing the market-share of ACTs in the private sector [20]. According to their evaluation, in mainland Tanzania the median price of quality-assured ACTs in the private for-profit sector decreased from 5.28 USD per adult equivalent treatment dose at baseline in 2010 to 0.94 USD (2010 USD) after AMFm implementation in 2011, identical to the endline price of SP, the most widely purchased antimalarial at that time [20]. Findings from this study are concerning in that, even with the success of the ACT subsidy by these measures, the majority of drug shop clients with malaria did not purchase ACTs in either region.

In addition, this study provides a basic assessment of how well the subsidy may be utilized in the private sector in the absence of diagnostic testing. With over 70% of ACTs purchased going to clients who tested negative for malaria, this study highlights the need for better targeting of ACTs. Studies in other settings have shown that drug shop staff and community members are interested in diagnostic testing and in RDTs becoming available at this level [10],[35]–[38]. Initial piloting of subsidized RDTs in drug shops in Uganda in 2011 found that RDT quality, sales, and correct usage were all maintained six months after drug shop attendants were trained on their use, though notably ACT purchase amongst persons who were RDT positive for malaria in that study was still just 32% [22]. Experience with subsidized RDTs in the private sector in Cambodia has also highlighted challenges in terms of poor uptake by consumers, and the need to structure the relative pricing of RDTs and ACTs to incentivize appropriate case management [39].

Challenges with malaria case management, even after the introduction of RDTs, have also been seen in the public sector. Multiple studies in Tanzania have shown that implementing policies requiring parasitological confirmation of malaria and training healthcare workers on RDT use significantly decreases the inappropriate prescription of antimalarial therapy, often times associated with a corresponding rise in antibiotic prescription [32] [40]–[43]. One recent study in three regions in Tanzania (including Mtwara and Mwanza) reported that even after RDT’s were introduced in the public sector, only 55% of febrile patients underwent laboratory testing for malaria and only 50% of patients positive for malaria (by exit-interview blood film) obtained an ACT [32]. Many questions remain about where private sector diagnostic testing should be implemented, how it can best be introduced (training, supervision, community messaging), how these shops should be monitored, and what unintended consequences might result. On-going pilots in Ghana, Nigeria, Uganda and Zambia may provide the data that are needed to determine where and how this can intervention can best be implemented (Unpublished data/on-going trials).

Since this study was performed, Tanzania has completed the expansion of the ADDO program to all regions. In addition, in late 2012, the Global Fund discontinued its separate funding stream for AMFm, and integrated the program into its core grant management and financial processes. Countries are now responsible for deciding whether or not to allocate Global Fund grants to private sector ACT subsidies. Decisions will also need to be made on whether or not to further initiate diagnostic testing in the private sector as a complementary measure. The Tanzanian government decided to continue with ACT subsidies, and to pilot the introduction of subsidized and low-cost (price-negotiated) RDTs in ADDOs in limited districts, in order to address the poor targeting of ACTs highlighted in this paper. This may potentially serve as a model for other countries that seek to support targeted and appropriate malaria care in the private sector within a limited-resource context.

## Conclusion

This study assessed the relationship between laboratory or parasitologically-confirmed malaria and ACT purchase in the private sector in the setting of a national ACT subsidy program and sub-national drug-shop training program. It showed that a substantial proportion of patients presenting to drug shops have malaria, and that even in the context of a national ACT subsidy program, less than a third of persons with malaria obtained ACTs in the private sector. It also showed that less than a quarter of persons obtaining ACTs tested positive for malaria. Expanding drug shop training and considering other measures of targeting ACTs, such as making diagnostic tests available in drug shops, should be considered to further improve access to and reduce inappropriate use of essential malaria medicines in the private sector in the future.
